# The Structure of Chaos: An Empirical Comparison of Fractal Physiology Complexity Indices Using NeuroKit2

**DOI:** 10.3390/e24081036

**Published:** 2022-07-27

**Authors:** Dominique Makowski, An Shu Te, Tam Pham, Zen Juen Lau, S. H. Annabel Chen

**Affiliations:** 1 School of Social Sciences, Nanyang Technological University, Singapore 639818, Singapore; teanshu97@gmail.com (A.S.T.); phamttam17@gmail.com (T.P.); lauzenjuen@gmail.com (Z.J.L.); 2 LKC Medicine, Nanyang Technological University, Singapore 639818, Singapore; 3 National Institute of Education, Nanyang Technological University, Singapore 637616, Singapore; 4 Centre for Research and Development in Learning, Nanyang Technological University, Singapore 639818, Singapore

**Keywords:** chaos, complexity, fractal, physiology, noise

## Abstract

Complexity quantification, through entropy, information theory and fractal dimension indices, is gaining a renewed traction in psychophsyiology, as new measures with promising qualities emerge from the computational and mathematical advances. Unfortunately, few studies compare the relationship and objective performance of the plethora of existing metrics, in turn hindering reproducibility, replicability, consistency, and clarity in the field. Using the NeuroKit2 Python software, we computed a list of 112 (predominantly used) complexity indices on signals varying in their characteristics (noise, length and frequency spectrum). We then systematically compared the indices by their computational weight, their representativeness of a multidimensional space of latent dimensions, and empirical proximity with other indices. Based on these considerations, we propose that a selection of 12 indices, together representing 85.97% of the total variance of all indices, might offer a parsimonious and complimentary choice in regards to the quantification of the complexity of time series. Our selection includes *CWPEn*, *Line Length (LL)*, *BubbEn*, *MSWPEn*, *MFDFA (Max)*, *Hjorth Complexity*, *SVDEn*, *MFDFA (Width)*, *MFDFA (Mean)*, *MFDFA (Peak)*, *MFDFA (Fluctuation)*, *AttEn*. Elements of consideration for alternative subsets are discussed, and data, analysis scripts and code for the figures are open-source.

## 1. Introduction

Complexity is an umbrella term for concepts derived from information theory, chaos theory, and fractal mathematics, used to quantify unpredictability, entropy, and/or randomness. Using these approaches to characterize physiological signals (a subfield commonly referred to as “fractal physiology” [1]) has shown promising results in the assessment and diagnostic of the state and health of living systems [2,3,4].

Over the past few decades, there has been an important increase in the number of complexity indices [5]. Although these new procedures are usually mathematically well-defined and theoretically promising, limited empirical evidence is available to understand their similarities and differences [2,5]. Moreover, some of these methods are resource-intensive and require long computation times. This complicates their application with techniques that utilise high sampling-rates (e.g., M/EEG) and makes them impractical to implement in real-time settings—such as brain-computer interfaces [6,7]. As such, having empirical data about the computation time of various complexity indices would prove useful, for instance to guide their selection, especially in contexts where time or computational resources are constrained.

Additionally, the lack of a comprehensive open-source and user-friendly software for computing various complexity indices likely contributes to the scarcity of empirical comparisons [8]. Indeed, many complexity indices are only described mathematically in journal articles, with reusable code seldom made available, therefore limiting their further application and validation [5,8]. Even when available and open-source, the code implementations of complexity measures are typically found scattered across different packages or scripts, or embedded within a larger goal-directed framework (e.g., *HCTSA*, a time-series comparison tool [9]). To address this lack of unified accessibility, we added a comprehensive set of complexity-related features to *NeuroKit2*, a Python package for physiological signal processing [10]. In doing so, we hope to provide users with an easy-to-use software capable of computing a wide range of complexity measures. The code is designed to run quickly and efficiently, while still being written in pure Python (with the help of standard dependencies such as *NumPy* or *Pandas* [11,12]) to maximize its reusability, transparency, and correctness.

Leveraging this tool, the goal of this study is to empirically compare a large number of complexity indices, inspect how they relate to one another, and derive recommendations for indices selection. More specifically, we will quantify the complexity of various types of signals with varying degrees of noise using 112 of the predominantly used indices that are available for computation with *NeuroKit2*. We will then project the results on a latent space through factor analysis, and review the indices that we find the most relevant and interesting in regards to their representation of the latent dimensions. This analysis will be complemented by hierarchical clustering. It should be noted that, even though this is one of the largest empirical comparison of complexity measures to date to our knowledge, the list of indices used is by no means exhaustive, with new indices constantly being developed, such as for instance *symmetropy* [13].

## 2. Methods

The Python script to generate the data can be found at https://github.com/DominiqueMakowski/ComplexityStructure (accessed on 21 July 2022).

**Figure 1:**
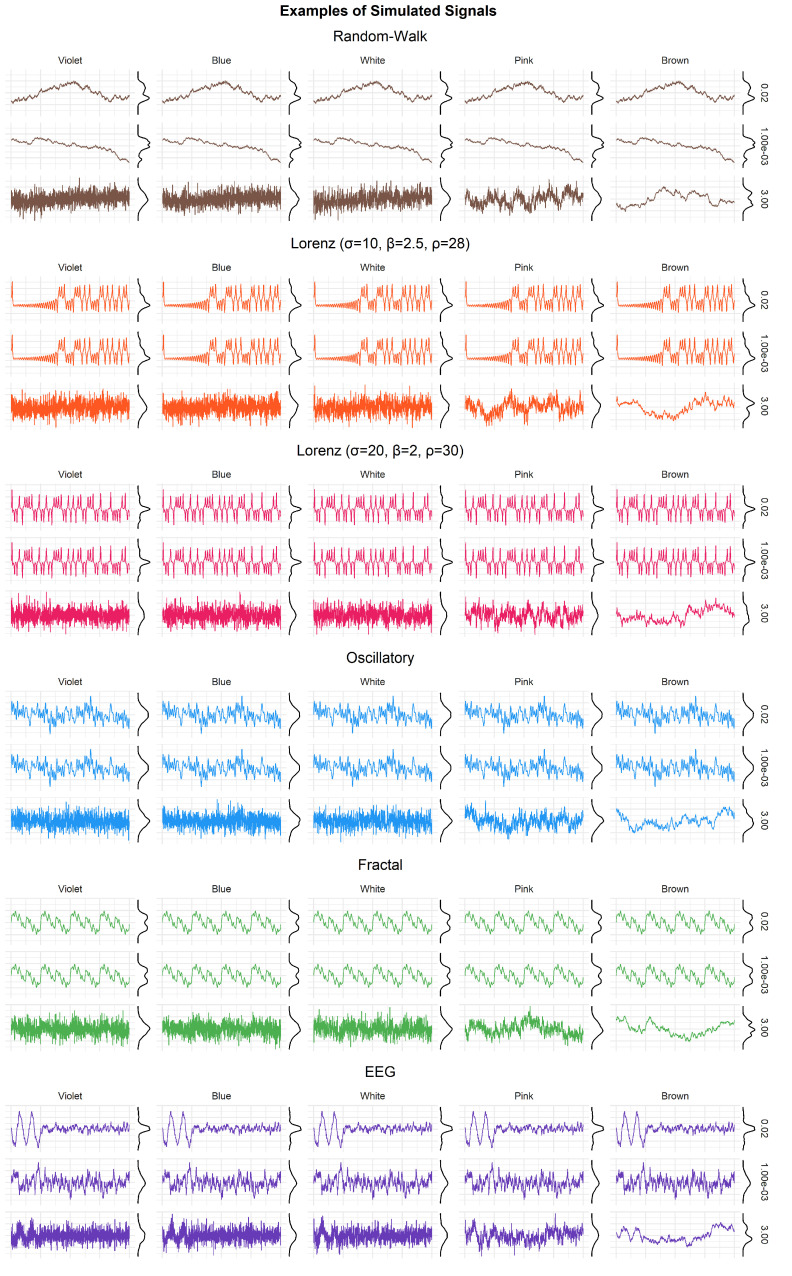
Different types of simulated signals, to which was added 5 types of noise (violet, blue, white, pink, and brown) with different intensities. For each signal type, the top row shows the signal with a minimal amount of noise, and the bottom row with a maximal amount of noise.

We started by generating 6 types of signals, one random-walk, two oscillatory signals (with one made of harmonic frequencies that results in a self-repeating - fractal-like - signal), two complex signals derived from Lorenz systems (with parameters σ=10, β=2.5, ρ=28; and σ=20, β=2, ρ=30, respectively) and one EEG-like simulated signal. Each of these signals was iteratively generated at 6 different lengths (ranging from 500 to 3000 by 500 samples). The resulting vectors were standardized and each were added 5 types of ${\left(1/f\right)}^{\beta }$ noise (namely violet β=−2, blue β=−1, white β=0, pink β=1, and brown β=2 noise). Each noise type was added at 128 different intensities (linearly ranging from 0.001 to 3). Examples of generated signals are presented in Figure 1.

The combination of these parameters resulted in a total of 23,040 signal iterations. For each of them, we computed 112 complexity indices, as well as additional basic metrics such as the *length* of the signal and its dominant *frequency*. We also included a *random* number at each iteration to ensure that our dimensionality analyses accurately discriminate this unrelated feature (as a manipulation check). The parameters used (such as the time-delay τ or the embedding dimension) are documented in the data generation script. For a complete description of the various indices included, please refer to NeuroKit’s documentation at https://neuropsychology.github.io/NeuroKit (accessed on 21 July 2022), in addition to the data generation script.

## 3. Results

The data analysis script and the data are fully available at https://github.com/DominiqueMakowski/ComplexityStructure (accessed on 21 July 2022). The analysis was performed in R using the *easystats* collection of packages [14,15,16,17]. As the results are primarily presented graphically via the figures, the code to fully reproduce them is also included in the analysis script.

### 3.1. Computation Time

**Figure 2:**
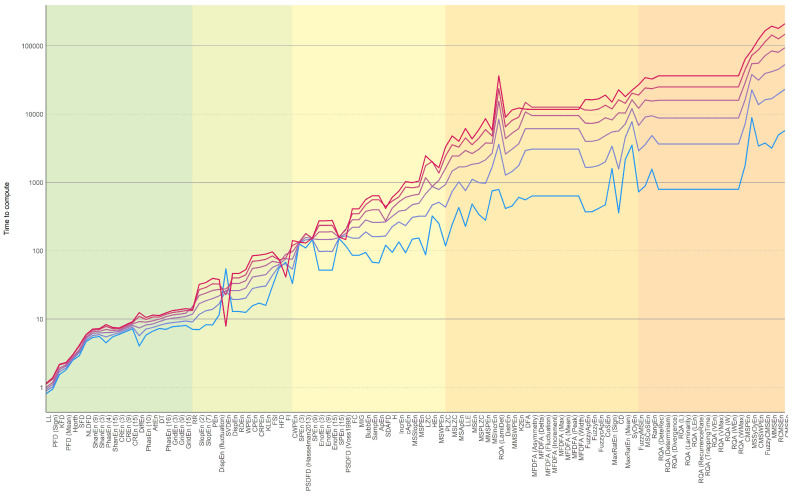
Median computation time difference between the different complexity indices algorithms, as well as variability as a function of signal lengths (represented by different line colors). The indices are grouped in sections (background color) according to their median computation time. Note that the time is expressed in arbitrary units as it is intended to convey differences, since the actual time would depend on the system specifications.

Firstly, one should note that the computation times presented in Figure 2 are relative (in arbitrary units) and do not correspond to real times, as these would highly depend on the system specifications. Rather, the goal here was to convey some intuition on the differences between different classes of indices (using the same machine and the same language of implementation, i.e., Python). While it is possible that computational advances or improvements in the code efficiency might change some of these values, we believe that the “big picture” should remain fairly stable, as it is to a large extent driven by the inherent nature of the algorithms under consideration.

Despite the relative shortness of the signals considered (a few thousand points at most), the fully-parallelized data generation script took around 24 h to run on a 48-cores machine. After summarizing and sorting the indices by computation time, the most striking result is the order of magnitude of difference between the fastest and slowest indices. Additionally, some indices are particularly sensitive to the signal length, a property which, in combination with their computational cost, led to indices being 100,000 times slower to compute than others.

In particular, multiscale indices were among the slowest to compute due to their iterative nature (a given index being computed multiple times on coarse-grained sub-series of the signal). Indices related to Recurrence Quantification Analysis (RQA) were also relatively slow and did not scale well with signal length.

For the subsequent analyses, we removed statistically redundant indices (which absolute correlation was equal to 1.0), such as *NLDFD*—identical to *LL*, *ShanEn (15)*—identical to *ShanEn (9)*, and *CREn (15)*—identical to *CREn (9)*. This results in a pool of 112 indices.

### 3.2. Correlation

**Figure 3:**
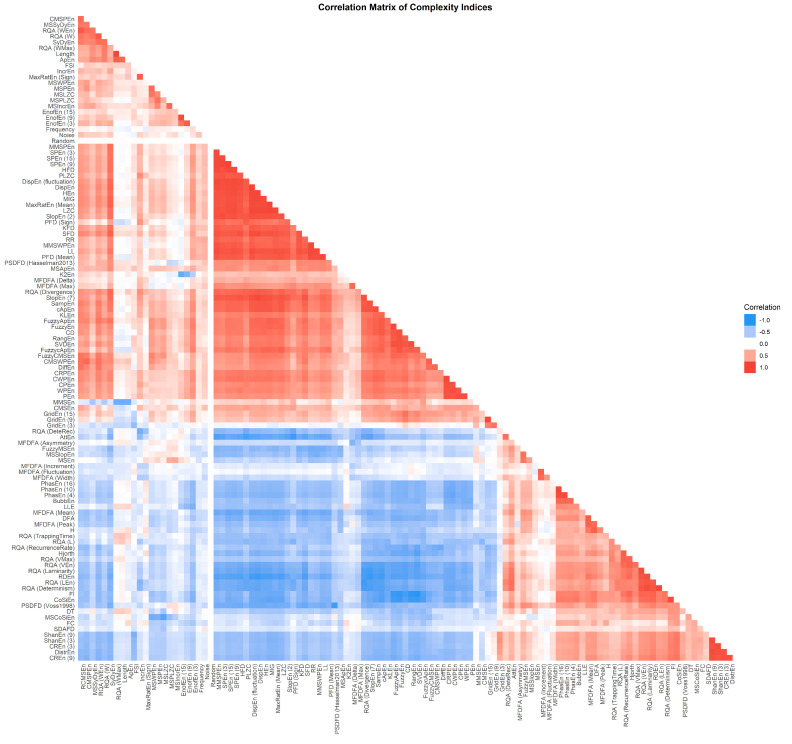
Correlation matrix of complexity indices.

The Pearson correlation analysis revealed that complexity indices, despite their multitude and their conceptual specificities, do indeed share similarities. They form two major clusters that are easily observable (the blue and the red groups in Figure 3). That being said, these two anti-correlated groups are mostly indicative of the fact that some indices, by design, index the “predictability”, whereas others, the “randomness”, and thus are negatively related to one another. In order to extract finer groupings, further analysis procedures were applied below.

### 3.3. Factor Analysis

**Figure 4:**
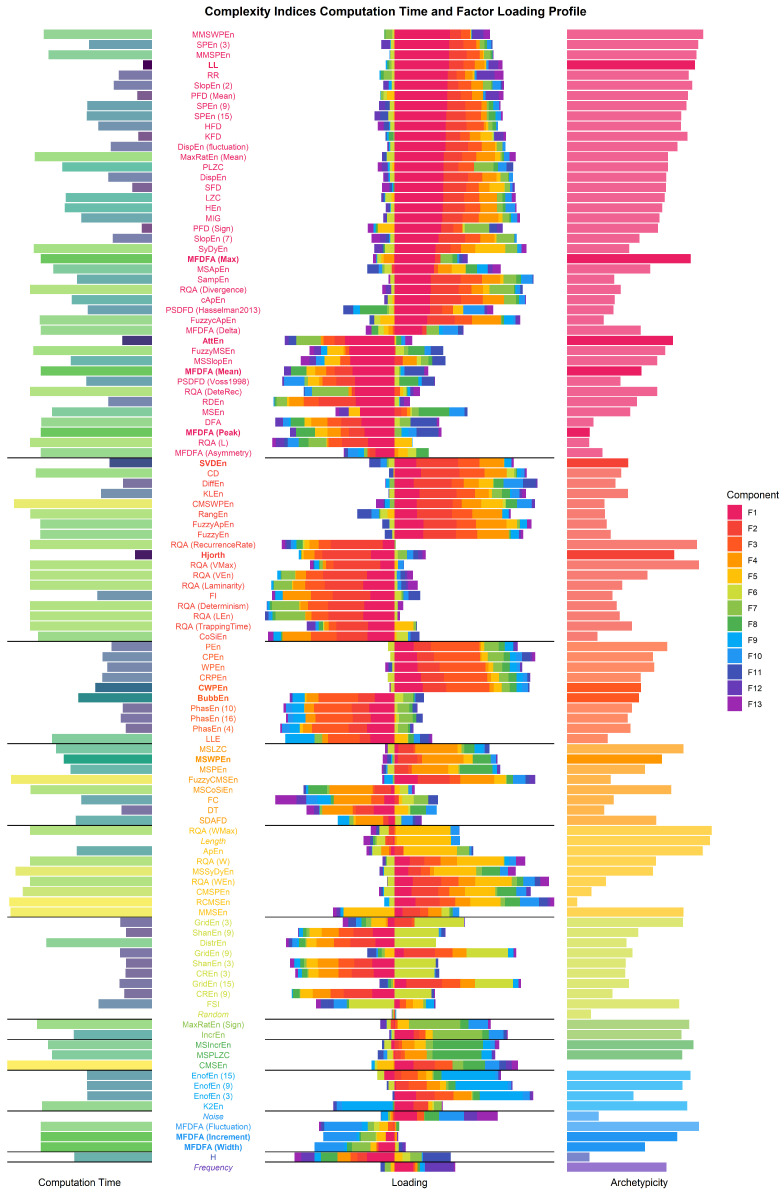
Factor loadings of the complexity indices, colored by the factor they represent the most (center). On the left, the median computation times and on the right, the archetypicity—the inverse of factor profile complexity (i.e., the extent to which each index is a pure representative of its dominant factor, which is low for indices that equally load on different factors).

The agreement procedure for the optimal number of factors suggested that the 112 indices can be mapped on a multidimensional space of 13 orthogonal latent factors, that we extracted using a *varimax* rotation. We then took interest in the loading profile of each index, and in particular the latent dimension that it maximally relates to (see Figure 4). Below are a description of the factors that we found to be interpretable.

The first extracted factor is the closest to the largest amount of indices, and is positively loaded by indices that are sensitive to the deviation of consecutive differences (e.g., *LL*, *PFD (Mean)*) as well as indices that capture the amplitude of fluctuations (*DispEn (fluctuation)*, *MFDFA (Max)*). In line with this, this factor was negatively loaded by indices related to Detrended Fluctuation Analysis (*DFA*), which tends to index the presence of long-term correlations and repetitions. As such, this latent factor might be associated with the predominance of short-term vs. long-term unpredictability.

The second factor was strongly loaded by indices that measure the feature-richness of the signal’s system (as most of them operate on a state-space decomposition). It was found to be positively related to *SVDEn* and the Kozachenko-Leonenko differential entropy (*KLEn*), and negatively to the RQA *Recurrence Rate* and *Hjorth* Complexity.

The third factor was loaded predominantly by permutation-based metrics (*PEn*, *WPEn*, *BubblEn*, etc.). The fourth factor included multiscale indices, such as *MSWPEn*. The fifth factor was strongly loaded by signal *length*, and thus might not capture features of complexity *per se*. Indices with the most relation to it were indices generally known to be sensitive to signal length, such as *ApEn*. The sixth factor was loaded by indices in which the signal or the Poincaré plot was discretized via binning or gridding, respectively. The seventh factor was loaded by sign-based entropy increments, and the eighth by multiscale *IncrEn* and multiscale *PLZC*. The ninth factor was loaded by *EnofEn* and Kolmogorov Entropy (*K2En*). The tenth factor was loaded positively by the amount of noise, and negatively by multifractal indices such as *MFDFA (Width)*, suggesting a sensitivity to regularity. Finally, as a manipulation check for our factorization method, the random vector did not load unto any factors.

### 3.4. Hierarchical Clustering and Connectivity Network

**Figure 5:**
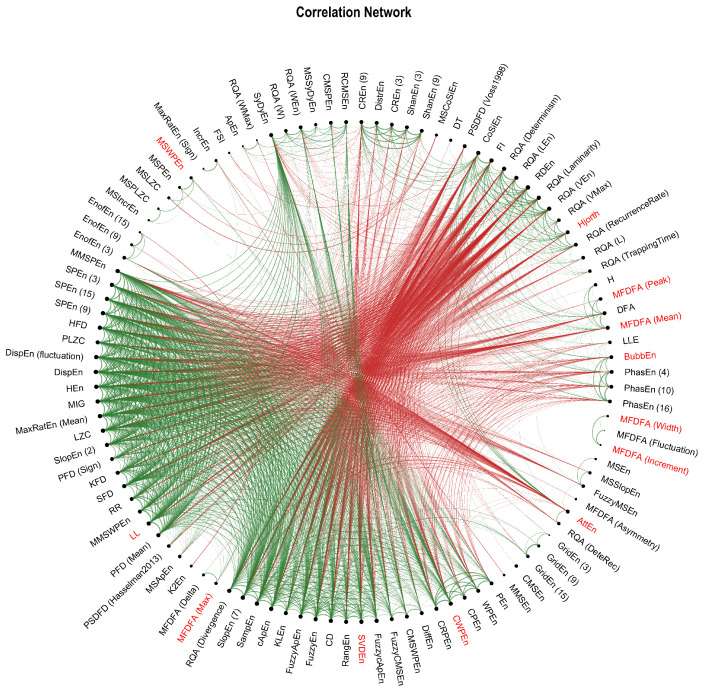
Correlation network of the complexity indices. Only the links where |r| > 0.6 are displayed.

**Figure 6:**
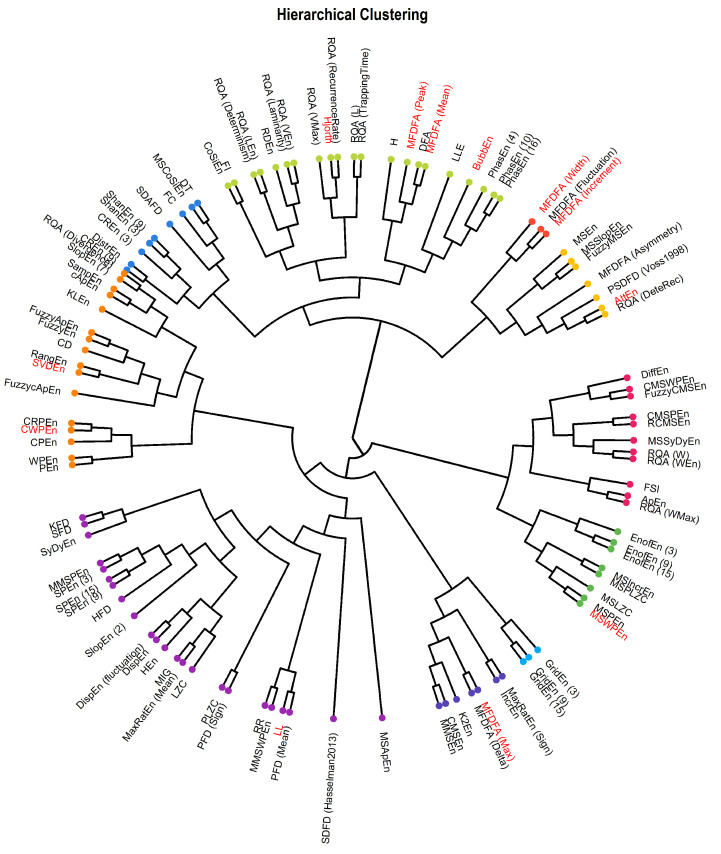
Dendrogram representing the hierarchical clustering of the complexity indices.

For illustration purposes, we represented the correlation matrix as a connectivity graph (see Figure 5). We then ran a hierarchical clustering (with a Ward D2 distance) to provide additional information about the groups discussed above. Indeed, while the factor analysis will predominantly show indices that are the most representative of a given latent dimension, clustering will construct groups based on the multidimensional profile (what dimensions a given index loads positively on, and what other does it load negatively on). This allowed us to refine our recommendations for complimentary complexity indices (see Figure 6).

### 3.5. Indices Selection

**Figure 7:**
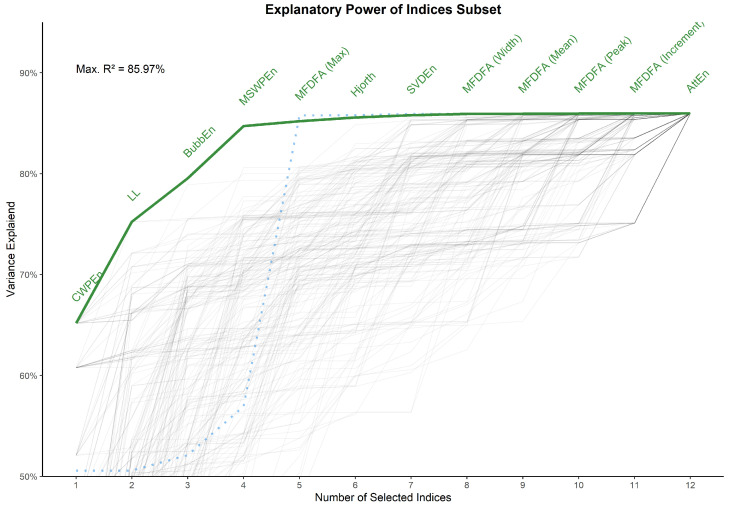
Variance of the whole dataset of indices explained by the subselection. Each line represents a random number of selected variables. The green line represents the optimal order (i.e., the relative importance) that maximizes the variance explained. The dotted blue line represents the cumulative relative median computation time of the selected indices, and shows that MFDFA and multiscale indices are the most resource-costly algorithms.

The selection of a subset of indices was based on a set of considerations: (1) high loadings on one predominant latent dimension, with additional attention to the pattern of secondary loadings. For instance, an index with a positive factor 1 loading and a negative factor 2 loading could complement another index with a similar factor 1 loading, but a positive factor 2 loading. This was facilitated by (2) the hierarchical clustering dendrogram (see Figure 6), with which we attempted to extract indices from each (meaningful) higher order clusters. Items related to clusters that we determine as being largely explained by noise, length or other artifacts were omitted. (3) A preference for indices with relatively shorter computation times. This yielded a selection of 12 indices. Next, we computed the cumulative variance explained by this selection in respect to the entirety of indices examined, and derived the optimal order to maximize the variance explained (see Figure 7). The 12 included indices, representing 85.97% of the variance of the whole dataset, were:

- *CWPEn*: The Conditional Weighted Permutation Entropy is based on the difference of weighted entropy between that obtained at an embedding dimension *m* and that obtained at m+1 [18].
- *LL*: The Line Length index stems out of a simplification of Katz’ fractal dimension (*KFD*) algorithm [19] and corresponds to the average of consecutive absolute differences. It is equivalent to *NDLFD*, the Fractal dimension via Normalized Length Density [20]. As it captures the amplitude 1-lag fluctuations, this index is likely sensitive to noise in the series.
- *BubbEn*: The Bubble Entropy is based on Permutation Entropy. It uses the *Bubble sort* algorithm and counts the number of swaps each vector undergoes in the embedding space instead of ranking their order [21].
- *MSWPEn*: The Multiscale Weighted Permutation Entropy is the entropy of weighted ordinal descriptors of the time-embedded signal computed at different scales obtained by a coarse-graining procedure [22].
- *MFDFA (Max)*: The value of singularity spectrum *D* corresponding to the maximum value of singularity exponent *H*.
- *Hjorth*: Hjorth’s Complexity is defined as the ratio of the mean frequency of the first derivative of the signal to the mean frequency of the signal [23].
- *SVDEn*: The Singular Value Decomposition (SVD) Entropy quantifies the amount of eigenvectors needed for an adequate representation of the system [24].
- *MFDFA (Width)*: The width of the multifractal singularity spectrum [25] obtained via Detrended Fluctuation Analysis (DFA).
- *MFDFA (Mean)*: The mean of the maximum and minimum values of singularity exponent *H*, which quantifies the average fluctuations of the signal.
- *MFDFA (Peak)*: The value of the singularity exponent *H* corresponding to peak of singularity dimension *D*. It is a measure of the self-affinity of the signal, and a high value is an indicator of high degree of correlation between the data points.
- *MFDFA (Increment)*: The cumulative function of the squared increments of the generalized Hurst’s exponents between consecutive moment orders [26].
- *AttEn*: The Attention Entropy is based on the frequency distribution of the intervals between the local maxima and minima of the time series [27].

**Figure 8:**
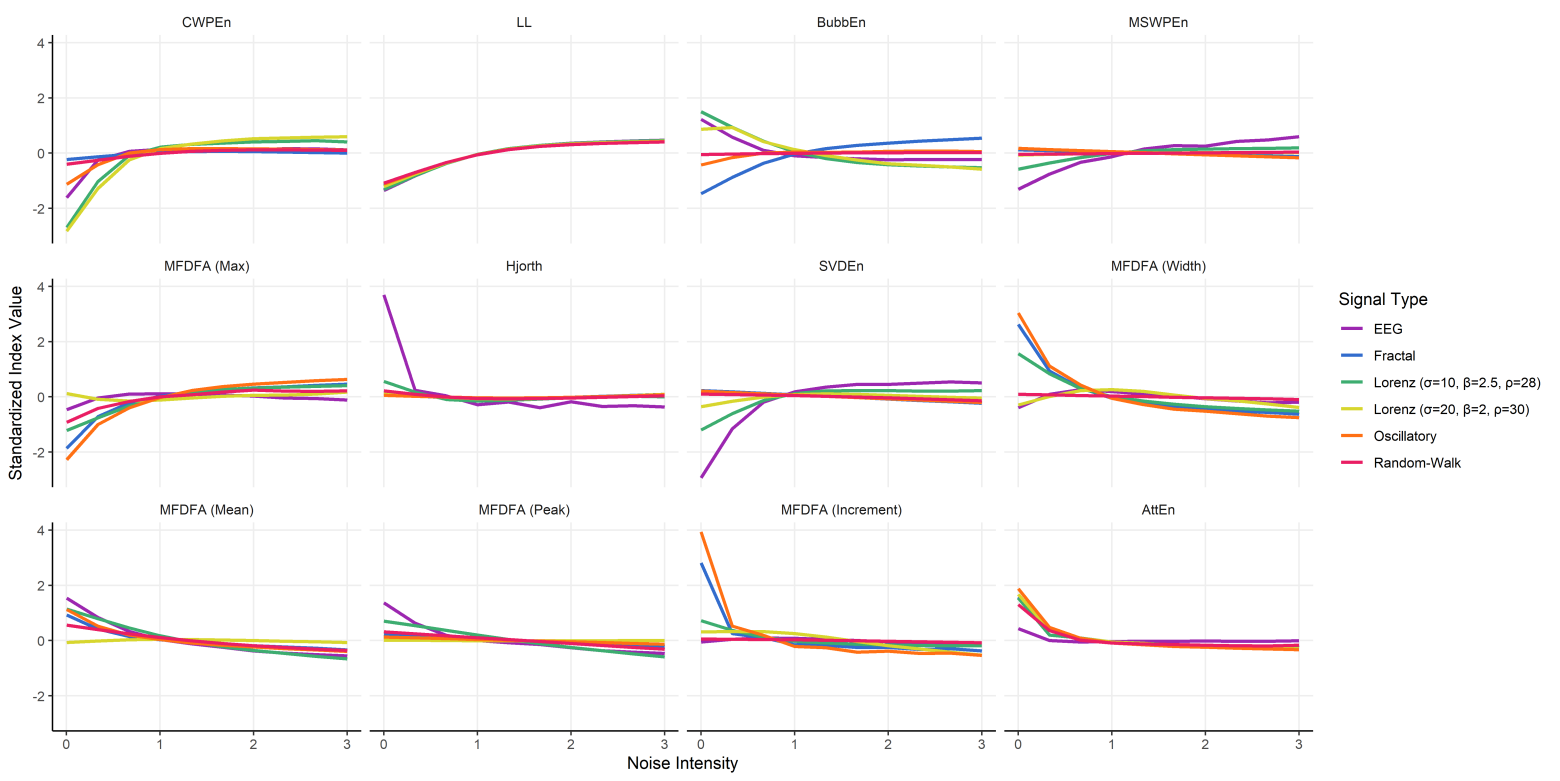
Visualization of the expected value of a selection of indices depending on the signal type and of the amount of noise.

Finally, we visualized the expected value of our selection of indices for different types of signals under different conditions of noise (see Figure 8). This confirmed that *LL* was primarily driven by the noise intensity (which is expected, as they capture the variability of successive differences). The other indices appear capable of discriminating between the various types of signals (when the signal is not dominated by noise).

## 4. Discussion

As the span and application of complexity science grows, a systematic approach to compare their “performance” becomes necessary to reinforce the clarity and structure of the field. The term *performance* used here is to be understood in a relative sense, as any such endeavor faces the “hard problem” of complexity science: various objective properties of signals (e.g., short-term vs. long-term variability, auto-correlation, information, randomness [28,29]) participate in forming overarching concepts such as “complex” and “chaotic”. Indices that are sensitive to some of these objective properties are thus conceptually linked through such overarching frameworks. However, it remains unclear how these high-level concepts transfer back, in a top-down fashion, into a combination of lower-level features. As such, it is conceptually complicated to benchmark complexity measures against “objectively” complex vs. non-complex signals. In other words, while we are aware that different objective signal characteristics can contribute to the “complexity” of a signal, there is not a one-to-one correspondence between the latter and the former.

To circumvent the aforementioned consideration, we adopted a paradigm where we generated different types of signals to which we systematically added distinct types—and amount—of perturbations. It should be noted that we did not seek to measure how complexity indices can discriminate between these signal types, nor did we attempt at mimicking real-life signals or scenarios. The goal was instead to generate enough variability to reliably map the relationships between the indices.

Our results empirically confirm the plurality of underlying components of complexity (although it is here defined somewhat circularly as what is measured by complexity indices), and more importantly show that complexity indices vary in their sensitivity to various orthogonal latent dimensions. However, the mostly descriptive interpretation of these dimensions is a limitation of the present investigation, and future studies are needed to investigate and discuss them in greater depth (for instance, by modulating specific properties of signals and measuring their impact on these latent dimensions).

Given the increasing role of complexity science as a field and the sheer number of complexity indices already published, our study aimed to empirically map the relationship between various indices and provide useful information to guide future researchers in their selection of complexity metrics. An example of indices subselection that encapsulates information about different underlying dimensions at a relatively low computational cost include *CWPEn*, *Line Length (LL)*, *BubbEn*, *MSWPEn*, *MFDFA (Max)*, *Hjorth Complexity*, *SVDEn*, *MFDFA (Width)*, *MFDFA (Mean)*, *MFDFA (Peak)*, *MFDFA (Fluctuation)*, *AttEn*. These indices might be complimentary in offering a parsimonious, yet comprehensive profile of the complexity of a time series. Moving forward, future studies are needed to validate, analyze and interpret the nature of the dominant sensitivities of various indices groups identified in the present work. In doing so, complexity findings in prospective studies can be more easily interpreted and integrated into new research and novel theories.

## Data Availability

https://github.com/DominiqueMakowski/ComplexityStructure (accessed on 21 July 2022).
